# Did a change in Nature journals’ editorial policy for life sciences research improve reporting?

**DOI:** 10.1136/bmjos-2017-000035

**Published:** 2019-02-26

**Authors:** The NPQIP Collaborative group

**Affiliations:** Centre for Clinical Brain Sciences, University of Edinburgh, Edinburgh, UK

**Keywords:** BMJOS, checklist, quality improvement

## Abstract

**Objective:**

To determine whether a change in editorial policy, including the implementation of a checklist, has been associated with improved reporting of measures which might reduce the risk of bias.

**Methods:**

The study protocol has been published at doi: 10.1007/s11192-016-1964-8.

**Design:**

Observational cohort study.

**Population:**

Articles describing research in the life sciences published in Nature journals, submitted after 1 May 2013.

**Intervention:**

Mandatory completion of a checklist during manuscript revision.

**Comparators:**

(1) Articles describing research in the life sciences published in Nature journals, submitted before May 2013; and (2) similar articles in other journals matched for date and topic.

**Primary outcome:**

The primary outcome is change in the proportion of Nature articles describing in vivo research published before and after May 2013 reporting the ‘Landis 4’ items (randomisation, blinding, sample size calculation and exclusions). We included 448 Nature Publishing Group (NPG) articles (223 published before May 2013, and 225 after) identified by an individual hired by NPG for this specific task, working to a standard procedure; and an independent investigator used PubMed ‘Related Citations’ to identify 448 non-NPG articles with a similar topic and date of publication from other journals; and then redacted all articles for time-sensitive information and journal name. Redacted articles were assessed by two trained reviewers against a 74-item checklist, with discrepancies resolved by a third.

**Results:**

394 NPG and 353 matching non-NPG articles described in vivo research. The number of NPG articles meeting all relevant Landis 4 criteria increased from 0/203 prior to May 2013 to 31/181 (16.4%) after (two-sample test for equality of proportions without continuity correction, Χ²=36.2, df=1, p=1.8×10^−9^). There was no change in the proportion of non-NPG articles meeting all relevant Landis 4 criteria (1/164 before, 1/189 after). There were more substantial improvements in the individual prevalences of reporting of randomisation, blinding, exclusions and sample size calculations for in vivo experiments, and less substantial improvements for in vitro experiments.

**Conclusion:**

There was an improvement in the reporting of risks of bias in in vivo research in NPG journals following a change in editorial policy, to a level that to our knowledge has not been previously observed. However, there remain opportunities for further improvement.

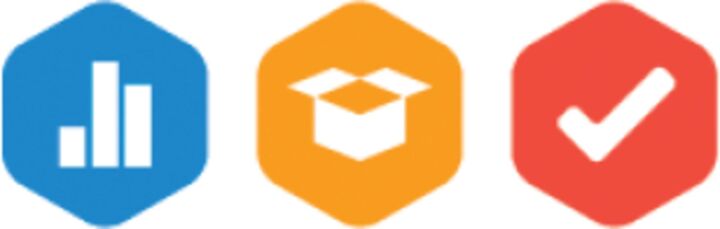

**Strengths and limitations of this study**

- Prospectively registered study with articulated protocol and statistical analysis plan, registered with the Open Science Framework.
- Study Dataset and code available in public domain.
- Involvement of a large collaborative group of outcome assessors.
- Articulation, in advance of analysis, of smallest effect size of interest.
- Retrospective observational study.
- Limited agreement between outcome assessors.

## Background

Few articles describing in vivo research report taking specific actions designed to reduce the risk that their findings are confounded by bias,^1^ and those that do not report such actions give inflated estimates of biological effects.^2 3^ Strategies and guidelines which might improve the quality of reports of in vivo research have been proposed,^4 5^ and while these have been endorsed by a large number of journals there is evidence that this endorsement has not been matched by a substantial increase in the quality of published reports.^6^ Poor replication of in vivo and in vitro research has been reported,^7–9^ and this has been attributed in part to poor descriptions of the experimental and analytical details.

In May 2013, Nature journals announced a change in editorial policy which required authors of submissions in the life sciences to complete a checklist, during manuscript revision, indicating whether or not they had taken certain measures which might reduce the risk of bias and to report key experimental and analytical details, and in their submission to detail where in the manuscript these issues were addressed.^10^ The development of this checklist was prompted in part by a consensus statement^5^ setting out key aspects of study design and conduct which were necessary to allow the reader to assess the validity of the findings presented; it identified these as randomisation, blinding, sample size estimation and data handling (the ‘Landis 4’). The Nature journals’ checklist also included items relating to figures and statistical representation of data; reagents used; species, strain and sex of experimental animals; reporting of relevant ethical approvals; consent (for research involving human subjects); data deposition; and availability of any bespoke computer code. The full checklist is given in online supplementary appendix 1.

The aim of this study was to determine whether the implementation of this checklist for submissions has been associated with improved reporting of measures that might reduce the risk of bias. Because the Landis consensus statement drew attention to randomisation, blinding, sample size estimation and data handling as being the most important items to report, we chose the reporting of these as our primary measure of outcome. To establish whether any observed change in quality was simply a secular trend occurring across all journals, we matched each included publication with a publication in a similar subject area published at around the same time by a different publisher.

## Methods

The methods are described in detail in the published study protocol,^11^ and the data analysis plan and analysis code were articulated prior to database lock and registered on the Open Science Framework (DOI: 10.17605/OSF.IO/HC7FK). The complete study data set including PubMed IDentifiers (PMIDs) and data descriptors (but not, for copyright reasons, the source pdfs) of the included articles is available on Figshare (10.6084/m9.figshare.6226718).

In this observational cohort study, we aimed to determine whether the implementation of a checklist for submissions has been associated with improved reporting of measures which might reduce the risk of bias. To do this we assessed whether—in the view of trained assessors—manuscripts reported the details required by the checklist. Importantly, we did not have access to the checklists completed by the authors.

The study populations comprised (1) published articles accepted for publication in Nature journals which described research in the life sciences and which were submitted after 1 May 2013 (when the mandatory completion of a checklist at the stage of manuscript revision was introduced) and before 1 November 2014; (2) published articles accepted for publication in Nature journals in the months preceding May 2013 which describe research in the life sciences; and (3) articles from other journals matched for subject area and time of publication. We measured the change in the reporting of items included in the checklist.

### Identification of relevant articles

We included studies which described in vivo (articles that contain at least one non-human animal experiment, including rodents, flies, worms, zebrafish and so on) or in vitro research.

#### NPG articles

One individual was specifically employed by the Nature Publishing Group (NPG) to select studies which (1) described in vivo or in vitro research and (2) were published in *Nature*, *Nature Neurology*, *Nature Immunology*, *Nature Cell Biology*, *Nature Chemical Biology*, *Nature Biotechnology*, *Nature Methods*, *Nature Medicine* or *Nature Structural & Molecular Biology*. First, the individual identified papers accepted for publication with an initial submission date later than 1 May 2013. Beginning with the then-current issues (volume corresponding to year 2015), they worked backwards in time, ensuring the submission date was after 1 May 2013, collecting papers with the intention of identifying 40 Nature papers and 20 from each of the other 8 titles (ie, 200 papers in total) (‘Post intervention’ group). They then used a similar process to identify papers submitted for publication before 1 May 2013, matched for journal and for country of origin (based on the address of the corresponding author), starting with the May 2013 issue and working backwards, ensuring that the date of submission was after 1 May 2011 (‘pre-intervention’ group). We sought to match on country of origin because at that time there was considerable discussion of national and regional differences in the quality of research publications (on the basis of anecdote rather than experience), and in an attempt to balance ‘writing in a foreign language’ between the two groups. Where no match could be found with a submission date after 1 May 2011 (ie, in a 2-year period), then the non-matched postintervention publication was excluded from analysis and a replacement postintervention publication selected, as above. A matching preintervention publication was then identified, as described above. Articles describing research involving only human subjects were excluded. A Nature editorial administrator independent of publishing decisions reviewed articles selected against the inclusion criteria and found some (less than 10%) had been included incorrectly; they replaced these with manuscript pairs that they selected according to the inclusion algorithm described above. The published files corresponding to the publication pdfs (including the extended methods section, extended data and other supplementary materials) were used to generate pdfs for analysis. These were provided to a member of our research team (RM) at a different institution, who used Adobe Acrobat to redact information relating to author names or affiliations, dates, volumes or page numbers, and the reference list, to minimise awareness of outcome assessors to whether the manuscript was preintervention or postintervention.

#### Non-NPG articles

The same member of our research team (RM) was responsible for identifying matching articles in other journals. Using PubMed, she entered the NPG publication title to retrieve the relevant record. She then added the ‘related citations for PubMed’ result to the search builder. In the second line search field of the search builder, she searched for ‘Date of publication’ in the same calendar month and year and performed the search. In the results returned she started with the first result returned and established whether it was published in a participating NPG journal (given in bold in table 1). If it was not, she applied the study inclusion criteria (in vivo or in vitro research or both, as defined above), ensuring that there was a match on the in vivo/in vitro status between the index NPG publication and the non-NPG publication. Where these criteria were met, she selected the publication for the study and retrieved the pdf, through open access, online institutional subscription, interlibrary loan or by request from the authors. If the first related citation did not fulfil these criteria, she moved to the next, until an appropriate publication was found. If an appropriate publication was not found, she repeated these steps but with the date of publication used in the search extended by 1 month earlier and 1 month later. If this process did not identify an eligible publication, she again extended the search by a month in each direction and continued until a matching publication was found. She then recorded the difference in calendar months between the date of publication of the index NPG article and the date of publication of the matching non-NPG article. She then used Adobe Acrobat to redact information relating to author names or affiliations, dates, volumes or page numbers, and the reference list, to minimise awareness of outcome assessors to whether the manuscript was preintervention or postintervention. Having completed these tasks she played no further part in the study.

**Table 1:** Sources of articles included in the study

Journal	n
***Nature***	**89**
*PLoS One*	47
***Nature Neuroscience***	**45**
***Nature Medicine***	**44**
***Nature Immunology***	**44**
***Nature Cell Biology***	**44**
***Nature Methods***	**43**
***Nature Genetics***	**40**
***Nature Biotechnology***	**40**
***Nature Chemical Biology***	**35**
*Proceedings of the National Academy of Sciences of the United States of America*	24
***Nature Structural & Molecular Biology***	**19**
*Journal of Neuroscience*	19
*Journal of Biological Chemistry*	13
*Journal of Immunology*	13
*Developmental Biology*	9
*Neuron*	7
*Cell Reports*	7
*Journal of Virology*	7
*Blood*	6
*Immunity*	6
*Cell*	6
*Gastroenterology*	6
*PLoS Genetics*	5
*Circulation Research*	5
*Biochemical and Biophysical Research Communications*	5
*EMBO Journal*	4
*Biological Psychiatry*	4
*Biochimica et Biophysica Acta*	4
*FASEB Journal*	4
*Genome Research*	4
*Development*	4
*Human Molecular Genetics*	3
*American Journal of Pathology*	3
*Journal of Cell Science*	3
*Stem Cells and Development*	3
*Experimental Neurology*	3
*Journal of Neuroscience Methods*	3
*Molecular Cell*	3
*European Journal of Immunology*	3
*Journal of Pharmacology and Experimental Therapeutics*	3
*Journal of Neurophysiology*	3
*Journal of Neuroscience Research*	3
*Molecular Cancer Therapeutics*	3
*Journal of Neurochemistry*	2
*Frontiers in Neural Circuits*	2
*Current Biology*	2
*Journal of Clinical Investigation*	2
*Journal of Cellular Physiology*	2
*Carcinogenesis*	2
*Journal of Cellular Biochemistry*	2
*Journal of Bone and Mineral Research*	2
*Vaccine*	2
*Infection and Immunity*	2
*Immunology*	2
*Hippocampus*	2
*Experimental Cell Research*	2
*Genes & Development*	2
*FEBS Journal*	2
*Journal of Cell Biology*	2
*Neurobiology of Disease*	2
*Molecular Pharmacology*	2
*Biomaterials*	2
*Science*	2
*Molecular and Cellular Endocrinology*	2
*Arteriosclerosis, Thrombosis, and Vascular Biology*	2
*Cancer Cell*	2
*Molecular Brain*	2
*Molecular Biology of the Cell*	2
*Cardiovascular Research*	2
*Biomedical Materials*	1
*eLife*	1
*Autophagy*	1
*Disease Models & Mechanism*	1
*Emerging Infectious Diseases*	1
*Endocrine-Related Cancer*	1
*Diabetologia*	1
*Eukaryotic Cell*	1
*Biochemistry*	1
*Biochemical Journal*	1
*BioMedical Engineering Online*	1
*Experimental Hematology*	1
*Food and Chemical Toxicology*	1
*Acta Physiologica (Oxford)*	1
*FEBS Letters*	1
*American Journal of Physiology-Heart and Circulatory Physiology*	1
*American Journal of Sports Medicine*	1
*Aquatic Toxicology*	1
*Annals of Neurology*	1
*European Journal of Medicinal Chemistry*	1
*Antimicrobial Agents and Chemotherapy*	1
*European Urology*	1
*European Journal of Pharmacology*	1
*Developmental Cell*	1
*European Journal of Neuroscience*	1
*Diabetes*	1
*Animal Genetics*	1
*Cellular Signalling*	1
*Cancer Prevention Research (Phila)*	1
*Cancer Research*	1
*International Journal of Developmental Biology*	1
*Cancer Immunology, Immunotherapy*	1
*Cell Cycle*	1
*Cell Growth & Differentiation*	1
*Cell Host & Microbe*	1
*Brain Structure and Function*	1
*Brain Stimulation*	1
*Brain Research*	1
*British Journal of Pharmacology*	1
*Cell Metabolism*	1
*Cellular Microbiology*	1
*Biophysical Journal*	1
*BMC Immunology*	1
*Development, Growth & Differentiation*	1
*Developmental Dynamics*	1
*Cancer Letters*	1
*Biotechnology and Bioengineering*	1
*BMC Bioinformatics*	1
*British Journal of Haematology*	1
*BMC Cell Biology*	1
*British Journal of Anaesthesia*	1
*Cytotherapy*	1
*BMC Physiology*	1
*Bone*	1
*Clinical Cancer Research*	1
*Circulation Journal*	1
*Bioorganic & Medicinal Chemistry Letters*	1
*BMC Cancer*	1
*Molecular Neurodegeneration*	1
*Mediators of Inflammation*	1
*Neuroscience*	1
*Neuropharmacology*	1
*Neuro-Oncology*	1
*Neoplasia*	1
*Mutagenesis*	1
*Molecular Vision*	1
*Nucleic Acids Research*	1
*Molecular Pain*	1
*Pharmacology Research & Perspectives*	1
*Molecular Immunology*	1
*Molecular Human Reproduction*	1
*Molecular Ecology*	1
*Molecular and Cellular Neuroscience*	1
*Molecular and Cellular Biology*	1
*Molecular BioSystems*	1
*Microbiology*	1
*International Immunopharmacology*	1
*Molecular Reproduction and Development*	1
*Reproductive Biology and Endocrinology*	1
*Toxicological Sciences*	1
*Tissue Engineering Part C: Methods*	1
*Tissue Engineering Part A*	1
*Stem Cells*	1
*Stem Cell Research*	1
*Stem Cell Reports*	1
*Science Translational Medicine*	1
*Nuclear Medicine and Biology*	1
*RNA*	1
*Journal of Zoo and Wildlife Medicine*	1
*Proteomics*	1
*Proteins*	1
*Prostate*	1
*Prostaglandins, Leukotrienes and Essential Fatty Acids*	1
*Proceedings of the Royal Society B: Biological Sciences*	1
*PLoS Pathogens*	1
*PLoS Biology*	1
*Physics in Medicine & Biology*	1
*Science Signaling*	1
*Hepatology*	1
*Microbial Pathogenesis*	1
*Investigative Ophthalmology & Visual Science*	1
*International Journal of Hematology*	1
*International Journal of Genomics*	1
*International Journal of Cancer*	1
*Acta Biomaterialia*	1
*Infection, Genetics and Evolution*	1
*Journal of Allergy and Clinical Immunology*	1
*Human Mutation*	1
*Journal of Applied Toxicology*	1
*Hearing Research*	1
*Gynecologic Oncology*	1
*Gut Microbes*	1
*Gut*	1
*Glia*	1
*Genetics & Epigenetics*	1
*Genes to Cells*	1
*Gene*	1
*Hypertension*	1
*Journal of Experimental Medicine*	1
*Journal of Surgical Research*	1
*Journal of Reproductive Immunology*	1
*Journal of Photochemistry and Photobiology B*	1
*Journal of Pharmaceutical and Biomedical Analysis*	1
*Journal of Pathology*	1
*Journal of Neural Engineering*	1
*Journal of Infectious Diseases*	1
*IUBMB Life*	1
*Journal of General Physiology*	1
*G3 (Bethesda)*	1
*Journal of Dental Research*	1
*Journal of Controlled Release*	1
*Journal of Comparative Neurology*	1
*Journal of Chromatography B Analytical Technologies in the Biomedical and Life Sciences*	1
*Journal of Bioscience and Bioengineering*	1
*Journal of Biomedical Optics*	1
*Journal of Biomedicine and Biotechnology*	1
*Journal of Autoimmunity*	1
*Journal of Hepatology*	1

We anticipated difficulty in identifying matching articles, and in particular in matching non-NPG articles by country; we did not seek to do so. In total 896 articles were selected for analysis.

### Outcome assessment

The Nature checklist focused on transparency in reporting and availability of materials and code, reflected in 10 items. We designed a series of questions (online supplementary appendix 2) to establish whether a given publication met or did not meet the requirements of the checklist. We did this to aid outcome assessors, because many checklist items included more than one embedded criteria. For instance, the section on ‘Figures and Statistical Representation of Data’ was operationalised to 12 individual ‘present/absent/not applicable’ responses. The checklist relates to the reporting of experiments, and so compliance could be achieved by reporting whether or not an element was described. For instance, for assessment of outcome, a publication was considered compliant if it reported that assessment was conducted blinded to experimental group, or if it reported that assessment was conducted without blinding to experimental group. A manuscript was only considered not to fulfil the requirements of the checklist if it described neither that the assessment was performed blinded to experimental group nor that the assessment was not performed blinded to experimental group.

Where a manuscript described both in vivo and in vitro research, the series of questions was completed for each. Where there was more than one in vitro experiment or more than one in vivo experiment, the question was considered in aggregate; that is, all in vitro experiments had to meet the requirements of the checklist item for the article to be considered compliant in reporting of in vitro experiments, and all in vivo experiments had to meet the requirements of the checklist item for the article to be considered compliant in reporting of in vivo experiments. Where an item was considered only partially compliant, we considered this, for the purposes of analysis, to be non-compliant. Where a particular checklist item was not relevant for a given manuscript (randomisation in observational studies, or power calculations in explicitly exploratory studies^12^), this item was considered ‘not applicable’ and the manuscript was not included in the analysis of that item.

Five researchers experienced in systematic review and risk of bias annotation scored a set of 10 articles using our series of questions. Disagreements were resolved by group discussion, to arrive at a set of ‘Gold standard’ answers for these 10 articles. We also used this experience to write a training guide for outcome assessors. We then used social media platforms and mailing lists to recruit outcome assessors. We sought to recruit individuals with a background in medicine or biomedicine at a graduate or undergraduate level who we believed should have experience in the critical appraisal of published materials. However, we also recruited two senior school students on Nuffield Research Placements in our group. After outcome assessors had reviewed the training materials, they were invited to score articles from the ‘Gold standard’ pool, presented in random order, until their concordance with the gold standard responses was 80% overall, and was 100% for the components of the primary outcome measure, for three successive articles. At this point we considered them to be trained. The 10 training data sets, with their ‘gold standard’ adjudications, were included in the analysis (using the gold standard adjudications). Because of the range of expertise available, we ensured that each manuscript was reviewed by at least one assessor highly experienced in systematic review and critical appraisal. The training platform remains available for continuing professional development at https://ecrf1.clinicaltrials.ed.ac.uk/npqip/.

PDF files of included articles were uploaded to the study website. Trained assessors were presented with articles for scoring in random order. Each manuscript was scored by two individuals, one with experience in systematic review and risks of bias annotation and one other. Disagreements between assessors were reconciled by a third, experienced individual who was not one of the original reviewers, who could see the responses previously given but not who were the initial reviewers. Each item for each manuscript was therefore scored by two (if there was agreement) or three (if there was disagreement) reviewers, except for the 10 manuscripts which served as the gold standard, which had been scored by five experienced assessors. We had intended to monitor outcome assessment after 10% of manuscripts had been scored and reconciled, but the reconciliation process lagged behind the outcome assessment, and this was not done.

### Statistical analysis plan

Given our focus on the reporting of measures to reduce the risks of bias, we took as our primary outcome measure a composite measure of the proportion of articles meeting the relevant measures identified by Landis *et al* in 2012 as being most important for transparency in reporting in vivo research. These are covered by items 2, 3 4 and 5 of the checklist and relate to the reporting of randomisation, of the blinded assessment of outcome, of sample size calculations, and of whether the manuscript described whether samples or animals were excluded from analysis. Importantly, checklist compliance did not require, for example, that the study was randomised, but rather that the authors stated whether or not it was randomised. The evaluation principle was to determine if someone with reasonable domain knowledge could understand the parameters of experimental design sufficiently to inform interpretation. It has been argued that these measures might not be as relevant for exploratory studies, and for these we recorded the item as ‘not relevant’. We defined exploratory studies as those where hypothesis testing inferential statistical analyses were not reported. Where an item was not relevant for a publication (for instance with studies using transgenic animals where group allocation had been achieved by Mendelian randomisation), we considered compliance as meeting all of the relevant criteria. Where a publication described both in vivo and in vitro experiments, we analysed each type of experiment separately.

Our primary outcome was the proportion of articles describing in vivo experiments published by NPG after May 2013 that meet all of the relevant Landis 4 criteria. This is described in the statistical analysis plan deposited on the Open Science Framework (osf.io/hc7fk) on 7 June 2017 prior to database lock and before we had derived any outcome information. Following discussion with the NPG editorial team, we also set out in the protocol^11^ some predefined ‘editorially significant changes’—either reaching compliance of 80% or an increase of 15% in compliance.

We used the two-sample proportion test (prop.test) in R without the Yates continuity correction and two-sided hypothesis testing to be sensitive to the possibility that performance might have declined rather than improved. The secondary outcomes were (1) whether the proportion of articles describing in vivo experiments published by NPG after May 2013 which met all four of the Landis 4 criteria was 80% or higher (the original primary outcome; Wald test, wald.ptheor.test, RVAideMemoire in R); (2) the change in the proportion of articles describing in vitro experiments published by NPG before and after May 2013 which met all four of the Landis 4 criteria (two-sample proportion test as above); and (3) the change in the proportion of manuscripts meeting the criteria for adequate reporting of statistical analysis details, individual Landis criteria, descriptions of animals, reagents and their availability, biological sequences or structures, computer code deposition, and items relating to the involvement of human subjects or materials in included studies. For the matching articles from non-NPG journals, the secondary outcomes were (1) the change in the proportion of articles describing in vivo experiments published before and after May 2013 which met all of the Landis 4 criteria (two-sample proportion test); (2) whether the proportion of articles describing in vivo experiments published after May 2013 which met all four of the Landis 4 criteria was 80% or higher (Wald test); (3) the change in the proportion of articles describing in vitro experiments published before and after May 2013 which met all four of the Landis 4 criteria (two-sample proportion test); and (4) the change in the proportion of manuscripts meeting the criteria for reporting of statistical analysis details, individual Landis criteria, descriptions of animals, reagents and their availability, biological sequences or structures, computer code deposition, and items relating to the involvement of human subjects or materials in included studies. For each of these outcomes, we compared the changes observed in NPG articles with that observed in non-NPG articles. For each secondary analysis we used the Holm-Bonferroni correction using the p.adjust option for prop.test in R to account for the number of comparisons drawn, as described in appendix B of the data analysis plan. We also used interrupted time series analysis for each checklist item to distinguish a discrete ‘shift’ in performance from an upward ‘drift’, as described in the data analysis plan. Several tertiary outcomes are described in the study protocol and statistical analysis plan and are reported in the supplementary material.

### Power calculations

Power calculations were performed in STATA (Version 13.0) prior to commencement of the study. For the primary outcome measure, we approximated required sample sizes using power calculations for a one-sided two-sample χ^2^ test in STATA seeking a significance level of p<0.01 and with varying estimates of compliance with the Landis 4 criteria in the preintervention group. With 200 articles in each group, we had 80% power to detect an increase from 10% to 21%, or from 20% to 34%, or from 30% to 45%, or from 40% to 56%, or from 50% to 66%. We wanted to detect an absolute difference of 10% or more and thought that compliance with the Landis 4 criteria in the preintervention group would be around 10%, so we thought that having 200 studies in each group would be enough.

For the primary outcome measure proposed in the original study protocol (that compliance with the Landis 4 criteria in the postintervention group reached 80%), 200 studies in each group would be sufficient to reject the alternative hypothesis if the observed compliance was 72% or lower, and again we considered this to be sufficient.

For individual checklist items, after correcting for multiple comparisons, statistical power again depends on the level of reporting in the preintervention group. Where this was between 15% and 85%, with 200 studies per group, we would have 80% power to detect an absolute increase of 15% in the reporting of each item. We considered this to be the minimal increase that would represent an important improvement in reporting. The power calculations are described in greater detail in the study protocol.^11^

## Results

**Figure 1:**
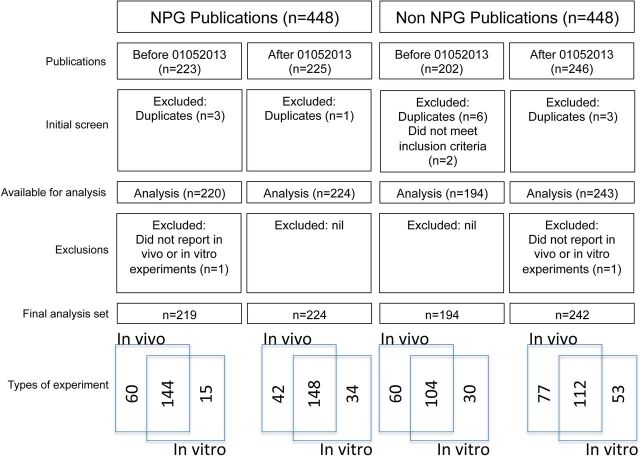
Articles initially included, and reasons for exclusion, and type of experiments described. NPG, Nature Publishing Group.

Eight hundred and ninety-six articles were identified and uploaded for outcome ascertainment, 448 in each cohort. Two non-NPG articles were excluded because they did not meet the inclusion criteria, and we identified four NPG and nine non-NPG articles which had been included more than once. Four hundred and forty-four NPG articles and 437 non-NPG articles underwent outcome assessment. One NPG publication and one non-NPG publication were adjudged at the time of outcome assessment to report neither in vivo nor in vitro research, and so were excluded. The analysis is therefore based on 443 NPG articles (219 before and 224 after 1 May 2013) and 436 non-NPG articles (194 before and 242 after 1 May 2013) (figure 1). The difference in numbers for NPG and non-NPG before and after 1 May 2013 is because some of the NPG ‘before’ articles matched best with articles in other journals published in the few months following May 2013. Specifically, 26 NPG preintervention articles were matched with other papers published an average of 3.2 months after May 2013 (maximum of 8 months), and 6 NPG postintervention articles were matched with other papers published 1, 2, 9, 11, 12 and 215 months before May 2013. Overall, 43% of matched pairs had dates of publication within 1 month, 54% within 2 months, 64% within 3 months and 81% within 6 months of each other (range −11 to +22 months). Two hundred and thirty-nine articles described only in vivo research, 133 described only in vitro research and 507 described both. Four hundred and ninety-four papers were completely matched for in vivo and in vitro status, 276 were partially matched (one member of matched pair reporting in vivo and in vitro research, the other reporting only in vitro or only in vivo research) and 36 were mismatched (one reporting only in vivo research, the other reporting only in vitro research). The source journals are given in table 1; in total 198 different titles contributed matching articles (median of 1 article per source journal, range 1–47). The PMIDs of included articles are listed in the data supplement.

We had intended to perform subgroup analyses in groups defined by country of origin, categorisation of research, and whether the study was predominantly in silico, in vitro, in vivo or involved human subjects. However, because for some countries the number of included manuscripts was low, the categorisation of research was not available for all manuscripts in the matching non-NPG group (except by inference from the matched NPG papers), the number of predominantly in silico and predominantly human studies was low, and we were not confident that we could operationalise a judgement as to whether a paper was predominantly in vivo or in vitro, we elected not to pursue these analyses. The country of origin for papers in each cohort is shown in table 2.

**Table 2:** Distribution of country of origin of the senior author in each cohort

Country	NPG (before)		NPG (after)		Non-NPG (before)		Non-NPG (after)		Total	
	n	%	n	%	n	%	n	%	n	%
USA	153	69.9	159	71.0	77	39.7	103	42.6	492	56.0
Germany	21	9.6	17	7.6	18	9.3	18	7.4	74	8.4
UK	14	6.4	16	7.1	5	2.6	19	7.9	54	6.1
China	5	2.3	4	1.8	15	7.7	20	8.3	44	5.0
Japan	3	1.4	3	1.3	25	12.9	10	4.1	41	4.7
Canada	4	1.8	3	1.3	7	3.6	13	5.4	27	3.1
France	5	2.3	6	2.7	7	3.6	5	2.1	23	2.6
Italy	3	1.4	3	1.3	2	1.0	7	2.9	15	1.7
South Korea					7	3.6	8	3.3	15	1.7
Switzerland	3	1.4	1	0.4	4	2.1	3	1.2	11	1.3
Australia	1	0.5	1	0.4	5	2.6	2	0.8	9	1.0
Spain	1	0.5	1	0.4	1	0.5	6	2.5	9	1.0
Austria	3	1.4	3	1.3			2	0.8	8	0.9
Sweden					6	3.1	2	0.8	8	0.9
The Netherlands	1	0.5			3	1.5	2	0.8	6	0.7
Taiwan			1	0.4	2	1.0	3	1.2	6	0.7
Denmark	1	0.5	2	0.9	1	0.5	1	0.4	5	0.6
India	1	0.5	1	0.4			3	1.2	5	0.6
Belgium					2	1.0	1	0.4	3	0.3
New Zealand					2	1.0	1	0.4	3	0.3
Finland					1	0.5	1	0.4	2	0.2
Hungary							2	0.8	2	0.2
Israel					1	0.5	1	0.4	2	0.2
Portugal					1	0.5	1	0.4	2	0.2
Singapore			1	0.4			1	0.4	2	0.2
Argentina							1	0.4	1	0.1
Bangladesh					1	0.5			1	0.1
Brazil							1	0.4	1	0.1
Bulgaria							1	0.4	1	0.1
Cameroon							1	0.4	1	0.1
Chile					1	0.5			1	0.1
Greece			1	0.4					1	0.1
Mexico							1	0.4	1	0.1
Norway			1	0.4					1	0.1
Poland							1	0.4	1	0.1
Russia							1	0.4	1	0.1
Total	219		224		194		242		879	

NPG, Nature Publishing Group.

Two hundred and five individuals registered with the project, of whom 109 started at least one training manuscript, 38 completed their training and 35 assessed at least one manuscript. Of these 35, 12 individuals also reconciled conflicting outcome assessments, and the web interface was programmed to ensure that they were not offered for reconciliation articles that they had previously adjudicated. Including reconciliation, the median number of articles scored was 13 (range 1–441). The agreement between outcome assessors ranged from being no better than chance at 50% (in vivo studies; implementation of statistical methods and measures: ‘Is the variance similar (difference less than two-fold) between the groups that are being statistically compared?’) to 98% (in vivo studies; ‘Does the study report the species?’). The median agreement was 82% (IQR 68%–89%). Two articles were identified during manuscript preparation as having been incorrectly recorded at data lock as reporting both in vivo and in vitro research, where in fact they only reported in vitro research, and one article had been incorrectly recorded as reporting both in vivo and in vitro research, where in fact it only reported in vivo research.

### Reporting of the Landis 4 items

The proportion of NPG in vivo studies reaching full compliance with the Landis 4 criteria increased from 0% (0/203) to 16.4% (31/189) (Χ²=36.1, df=1, p=1.8×10^−9^), but remained significantly lower than the target of 80% (95% CI 11.6% to 22.6%, Wald test versus 80% z=−15.4, p=2.2×10^−16^). In the tables the denominator number of studies (‘N’) differs according to whether that criterion is relevant to the work presented; for instance, in transgenic studies randomisation may not be appropriate (tables 3–5).

**Table 3:** Primary outcome: compliance with Landis 4 guidelines, in vivo research

Item	NPG before				NPG after					Non-NPG before				Non-NPG after				
	n	N	%	CI	n	N	%	CI	p	n	N	%	CI	n	N	%	CI	p
In vivo																		
Full Landis	0	203	0	0.0 to 2.3	31	189	16.4	11.6 to 22.6	<10^−8^	1	164	0.6	0.1 to 4.2	1	189	0.5	0.1 to 3.7	NS

n, number meeting the criteria; N, total number of studies where that criteria is relevant; %, per cent meeting the criteria; CI, 95% CI of that percentage; p, significance level (two-sample proportion test); NS, not significant at p<0.05.

NPG, Nature Publishing Group.

**Table 4:** Secondary outcome: full Landis compliance, in vitro research

Item	NPG before				NPG after				Adj p	Non-NPG before				Non-NPG after				Adj p
	n	N	%	CI	n	N	%	CI		n	N	%	CI	n	N	%	CI	
In vitro																		
Full Landis	0	159	0.0	0.0 to 2.3	6	182	3.3	1.5 to 7.1	NS	0	133	0.0	0.0 to 3.5	1	165	0.6	0.1 to 4.2	NS

n, number meeting the criteria; N, total number of studies where that criteria is relevant; %, per cent meeting the criteria; CI, 95% CI of that percentage; Adj p, adjusted significance level (two-sample proportion test (prop.test) followed by Holm-Bonferroni correction (p.adjust.methods)); NS, not significant at p<0.05.

NPG, Nature Publishing Group.

**Table 5:** Compliance with individual Landis 4 items, in vivo and in vitro research

Item	NPG before				NPG after				Adj p	Non-NPG before				Non-NPG after				Adj p
	n	N	%	CI	n	N	%	CI		n	N	%	CI	n	N	%	CI	
In vivo																		
Report method of randomisation?	3	170	1.8	0.6 to 5.3	19	170	11.2	7.2 to 16.9	NS	1	134	0.8	0.1 to 5.1	4	149	2.7	1.0 to 6.9	NS
Statement about randomisation	14	169	8.3	5.0 to 13.5	97	151	64.2	56.3 to 71.5	3×10^–14^	7	136	5.1	2.5 to 10.4	14	147	9.5	5.6 to 15.4	NS
Blinded?	8	198	4.0	2.0 to 7.9	42	184	22.8	17.3 to 29.4	8×10^–6^	2	162	1.2	0.3 to 4.8	7	183	3.8	1.8 to 7.8	NS
Statement about blinding	3	182	1.6	0.5 to 5.0	73	132	55.3	46.8 to 63.4	3×10^–14^	1	151	0.7	0 to 4.6	2	165	1.2	0 to 4.7	NS
Exclusions reported?	28	202	13.9	9.7 to 19.3	58	189	30.7	24.5 to 37.6	0.008	16	164	9.8	6.1 to 15.3	22	189	11.6	7.8 to 17.0	NS
Exclusion criteria defined?	24	200	12	8.2 to 17.3	35	188	18.6	13.7 to 24.8	NS	14	163	8.6	5.2 to 14.0	20	188	10.6	7.0 to 15.9	NS
Clear these were prespecified?	1	25	4	0.6 to 23.6	5	39	12.8	5.4 to 27.3	NS	0	17	0	0.0 to 22.9	0	21	0	0.0 to 19.2	NS
Was SSC done?	4	196	2.0	0.8 to 5.3	27	182	14.8	10.4 to 20.8	0.0008	0	156	0	0.0 to 3.0	3	183	1.6	0.5 to 5.0	NS
If not done, was SSC mentioned?	3	192	1.6	0.5 to 4.7	90	154	58.4	50.5 to 66.0	3×10^–14^	1	157	0.6	0 to 4.4	2	180	1.1	0.3 to 4.3	NS
In vitro																		
Report method of randomisation?	0	149	0	0.0 to 3.1	5	173	2.9	1.2 to 6.8	NS	1	125	0.8	0.1 to 5.5	1	157	0.6	0.1 to 4.4	NS
Statement about randomisation	0	149	0	0.0 to 3.1	26	167	15.6	10.8 to 21.9	7×10^–5^	0	123	0	0.0 to 3.8	4	156	2.6	1.0 to 6.6	NS
Blinded?	6	155	3.9	1.8 to 8.4	16	179	8.9	5.6 to 14.1	NS	3	131	2.3	0.7 to 6.9	1	162	0.6	0.1 to 4.2	NS
Statement about blinding	1	150	0.7	0.1 to 4.6	25	157	15.9	11.0 to 22.5	0.0002	0	127	0	0.0 to 3.7	1	158	0.6	0.1 to 4.3	NS
Exclusions reported?	13	159	8.2	4.8 to 13.6	29	182	15.9	11.3 to 22.0	NS	7	133	5.3	2.5 to 10.6	10	165	6.1	3.3 to 10.9	NS
Exclusion criteria defined?	12	159	7.5	4.3 to 12.8	23	178	12.9	8.7 to 18.7	NS	7	133	5.3	2.5 to 10.6	9	165	5.5	2.9 to 10.2	NS
Clear these were prespecified?	0	14	0	0.0 to 26.8	1	24	4.2	0.6 to 24.4	NS	0	8	0	0.0 to 40.0	0	11	0	0.0 to 32.1	NT
Was SSC done?	2	155	1.3	0.3 to 5.0	14	177	7.9	5.1 to 13.5	NS	0	129	0	0.0 to 3.6	0	161	0	0.0 to 2.9	NS
If not done, was Sample Size Calculation mentioned?	5	153	3.3	1.4 to 7.6	47	165	28.5	22.1 to 35.8	2×10^–7^	0	129	0	0.0 to 3.6	1	162	0.6	0.1 to 4.2	NS

n, number meeting the criteria; N, total number of studies where that criteria is relevant; %, per cent meeting the criteria; CI, 95% CI of that percentage: Adj p, adjusted significance level (two-sample proportion test (prop.test) followed by Holm-Bonferroni correction (p.adjust.methods)); NS, not significant at p<0.05: NT, not tested (n<10 for one of the comparisons).

NPG, Nature Publishing Group.

Because the number of manuscripts with a country of origin other than the USA and the number of studies which were predominantly in silico (we used studies which used computer code as a surrogate for the upper extent of this) were small, we did not analyse these further. There were no differences in compliance with the primary outcome measure dependent on whether the study included human research, or whether they included both in vivo and in vitro research or in vivo research alone.

#### Randomisation

**Figure 2:**
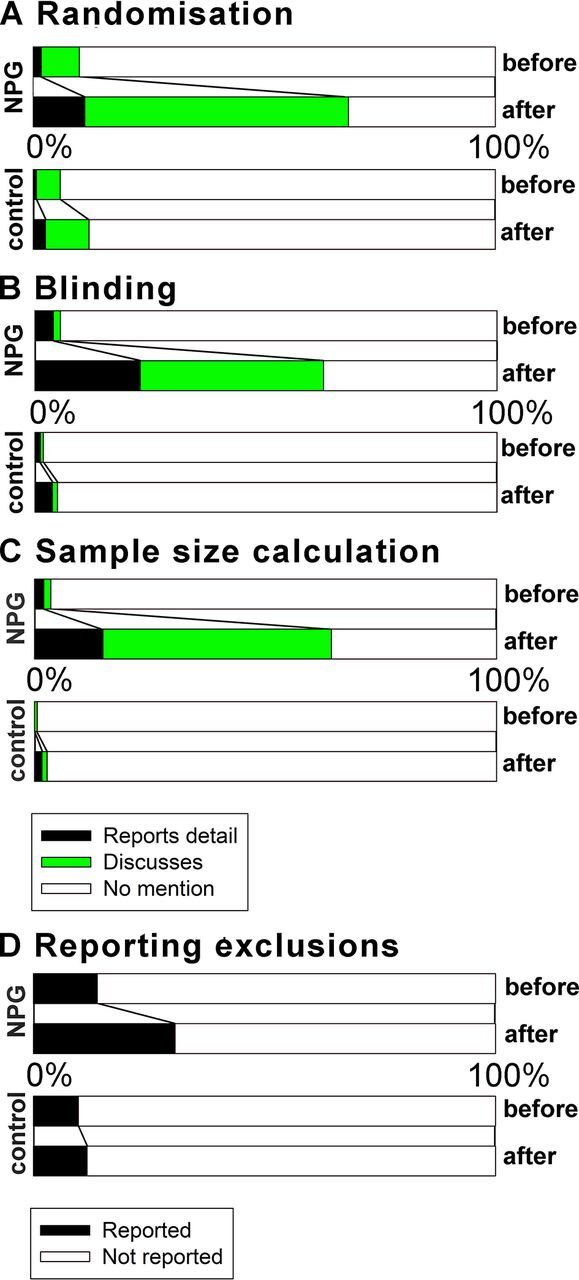
Compliance with each Landis criteria for in vivo experiments for NPG (top two panels of each quartet) and non-NPG articles (lower two panels) before and after 1 May 2013. (A) Randomisation; (B) blinding; (C) sample size calculation; and (D) reporting of exclusions. For A–C, black represents studies where compliance was achieved by reporting that the measure was taken; green that compliance was achieved by describing that the measure was not taken; and white that compliance was not achieved. For D, black represents studies where exclusions were reported and white that exclusions were not reported. NPG, Nature Publishing Group.

The preferred standard is that the manuscript describes which method of randomisation was used to determine how samples or animals were allocated to experimental groups, although articles were also compliant if they included a statement about randomisation even if no randomisation was used. The proportion of NPG in vivo studies reporting the method of randomisation was 1.8% before and 11.2% after (χ²=12.4, df=1, adjusted p=0.054). Of the remainder, the proportion of studies mentioning randomisation increased from 8.3% to 64.2% (χ²=110.2, df=1, adjusted p=3.2×10^−14^); overall, 68% of studies discussed randomisation in some way and so were judged compliant. Figure 2A shows change in the proportion of studies meeting these criteria before and after the change in editorial policy.

#### Blinding

The preferred standard is that the manuscript describes whether the investigator was blinded to the group allocation during the experiment and/or when assessing the outcome, although articles were also compliant if they included a statement about blinding even if no blinding was done. The proportion of NPG in vivo studies reporting blinding during group allocation or outcome assessment or both increased from 4% to 22.8% (Χ²=29.6, df=1, adjusted p=7.6×10^−6^). Of the remainder, the proportion of studies mentioning blinding increased from 1.6% to 55.3% (Χ²=120.1, df=1, adjusted p<3.2×10^−14^); overall, 63% of studies discussed blinding in some way and so were judged compliant. Figure 2B shows change in the proportion of studies meeting these criteria before and after the change in editorial policy.

#### Exclusions

The proportion of studies reporting animals excluded from analysis increased from 13.9% to 30.7% (Χ²=16.1, df=1, adjusted p=0.008). Figure 2C shows change in the proportion of studies meeting these criteria before and after the change in editorial policy.

#### Sample size calculations

The preferred standard is that the manuscript describes how the sample size was chosen to ensure adequate power to detect a prespecified effect size, although articles were also compliant if they included a statement about sample size estimate even if no statistical methods were used. The proportion of studies reporting an a priori sample size calculation increased from 2.0% to 14.8% (Χ²=20.5, df=1, adjusted p=0.0008). Of the remainder, the proportion of studies mentioning sample size calculations increased from 1.6% to 58.4% (Χ²=140.7, df=1, adjusted p<3.2×10^−14^); overall, 64% of studies discussed sample size calculations in some way and so were judged compliant. Figure 2D shows change in the proportion of studies meeting these criteria before and after the change in editorial policy.

For NPG in vitro studies, the proportion reaching full compliance with the Landis 4 criteria was 0% (0/159) before and 3.3% (6/176) after (Χ²=6.8, df=1, Holm-Bonferroni adjusted p=1.00). The proportion of studies reporting randomisation was 0% before and 2.9% after (Χ²=4.4, df=1, adjusted p=1.00). The proportion of studies mentioning randomisation even where it was not reported increased from 0% to 15.6% (Χ²=25.3, df=1, p=6.9×10^−5^). The proportion of studies reporting blinding during group allocation or outcome assessment or both was 3.9% before and 8.9% after (Χ²=3.467, df=1, p=1.00). The proportion of studies mentioning blinding even where it was not reported increased from 0.7% to 15.9% (Χ²=23.0, df=1, p=0.0002). The proportion of studies reporting exclusions from analysis was 8.2% before and 15.9% after (Χ²=4.73, df=1, p=1.00). The proportion of studies reporting an a priori sample size calculation was 1.3% before and 7.9% after (Χ²=8.7106, df=1, p=1.00). The proportion of studies mentioning sample size even where a sample size calculation was not reported increased from 3.3% to 28.5% (Χ²=36.9, df=1, p=1.8×10^−7^).

The proportion of matching (non-NPG) in vivo studies reaching full compliance with the Landis 4 criteria was 1% before and 1% after (Χ²=0.01, df=1, adjusted p=1.00), and for in vitro studies the proportion of non-NPG studies reaching full compliance with the Landis 4 criteria was 0% before and 1% after (Χ²=0.8, df=1, adjusted p=1.00). There was no significant change in reporting of any of the individual Landis 4 criteria for either in vivo or in vitro research.

### Statistical reporting

For in vivo studies reported in NPG articles, there were significant improvements in the reporting of exact numbers (from 46% to 69%, Χ²=22.07, df=1, adjusted p=0.0004), of whether t-tests were defined as one-sided or two-sided (from 46% to 71%, Χ²=17.80, df=1, adjusted p=0.003), and whether the assumptions of the test had been checked (from 9% to 27%, Χ²=18.58, df=1, adjusted p=0.002). For in vitro experiments described in NPG articles, there were significant improvements in the reporting of the exact numbers (from 32% to 70%, Χ²=12.60, df=1, adjusted p=0.05), of whether data represented technical or biological replicates (from 57% to 75%, Χ²=13.29, df=1, adjusted p=0.035), and whether t-tests were defined as one-sided or two-sided (from 47% to 72%, Χ²=16.18, df=1, adjusted p=0.008). For in vivo and in vitro studies described in non-NPG articles, there was no significant change in any of the items relating to statistical reporting (table 6).

**Table 6:** Secondary outcome: statistical items, in vivo and in vitro experiments

Item	NPG before				NPG after				Adj p	Non-NPG before				Non-NPG after				Adj p
	n	N	%	CI	n	N	%	CI		n	N	%	CI	n	N	%	CI	
In vivo																		
Exact n	93	203	45.8	39.8 to 52.7	131	189	69.3	62.4 to 75.5	0.0004	76	164	46.3	38.8 to 54.0	89	189	47.1	40.1 to 54.2	NS
Technical or biological replicates	137	201	68.2	61.4 to 74.2	151	188	80.3	74.0 to 85.4	NS	92	164	56.1	48.4 to 63.5	102	188	54.3	47.1 to 61.2	NS
Number of times replicated	45	199	22.6	17.3 to 28.9	68	183	37.2	30.5 to 44.4	NS	35	164	21.3	15.7 to 28.3	36	188	19.1	14.1 to 25.4	NS
Test described if uncommon?	26	51	51.0	37.5 to 64.3	41	68	60.3	48.3 to 71.7	NS	24	42	57.1	41.1 to 71.9	30	53	56.6	42.4 to 69.9	NS
t-Test defined as one-sided or two-sided?	61	133	45.9	37.6 to 54.4	102	144	70.8	62.9 to 77.7	0.003	30	100	30.0	21.8 to 39.7	50	109	45.9	36.8 to 55.3	NS
Correction for multiplicity	63	116	54.3	45.2 to 63.1	69	122	56.6	47.6 to 65.1	NS	57	101	56.4	46.6 to 65.8	74	118	62.7	53.7 to 71.0	NS
Reporting full statistics	39	167	23.4	17.6 to 30.4	36	171	21.1	15.6 to 27.8	NS	21	136	15.4	10.3 to 22.5	20	156	12.8	8.4 to 19.0	NS
Reporting of average	135	181	74.6	67.7 to 80.4	147	171	86.0	79.1 to 90.4	NS	110	147	74.8	67.2 to 81.2	121	168	72.0	64.8 to 78.3	NS
Definition of error bars	159	181	87.8	82.2 to 91.9	155	168	92.3	87.1 to 95.4	NS	118	144	81.9	74.8 to 87.4	129	165	78.2	71.2 to 83.8	NS
Testing of assumptions	15	171	8.8	5.4 to 14.0	44	165	26.7	20.5 to 33.9	0.002	12	134	9.0	5.2 to 15.1	18	156	11.5	7.4 to 17.6	NS
Reporting measures of variation	143	183	78.1	71.6 to 83.5	139	172	80.8	74.2 to 86.0	NS	115	142	81.0	73.4 to 86.9	125	169	74.0	66.6 to 80.3	NS
Variation less than twofold	13	53	24.5	14.8 to 37.8	22	61	36.1	25.1 to 48.8	NS	12	49	24.5	14.5 to 38.4	17	50	34.0	22.3 to 48.0	NS
In vitro																		
Exact n	51	158	32.3	25.4 to 40.0	93	181	70.5	44.1 to 58.6	0.050	49	133	36.8	29.1 to 45.4	43	165	26.1	19.9 to 33.3	NS
Technical or biological replicates	90	159	56.6	48.8 to 64.1	137	182	75.3	68.5 to 81.0	0.035	52	133	39.1	31.2 to 47.6	70	165	42.4	35.1 to 50.1	NS
Number of times replicated	62	158	39.2	31.9 to 47.1	85	181	47.0	39.8 to 54.2	NS	48	132	36.4	28.6 to 44.9	58	164	35.4	28.4 to 43.0	NS
Test described if uncommon?	23	42	54.8	39.7 to 69.0	36	58	62.1	49.0 to 73.6	NS	19	44	43.2	29.5 to 58.0	14	39	35.9	22.6 to 51.9	NS
t-Test defined as one-sided or two-sided?	51	109	46.8	37.6 to 56.2	94	130	72.3	64.0 to 79.3	0.008	27	82	32.9	23.6 to 43.8	30	89	33.7	24.7 to 44.1	NS
Correction for multiplicity	48	102	47.1	37.6 to 56.7	59	114	51.8	42.6 to 60.8	NS	40	95	42.1	36.7 to 57.6	54	100	54.0	44.2 to 63.5	NS
Reporting full statistics	25	140	17.9	12.4 to 25.1	27	160	16.9	11.8 to 23.5	NS	16	112	14.3	8.9 to 22.0	19	135	14.1	9.2 to 21.0	NS
Reporting of average	122	149	81.9	74.5 to 87.3	140	160	87.5	81.4 to 91.8	NS	90	126	71.4	62.9 to 78.6	111	148	75.0	67.4 to 81.3	NS
Definition of error bars	136	149	91.3	85.6 to 94.9	155	164	94.5	89.8 to 97.1	NS	98	122	80.3	72.3 to 86.4	119	146	81.5	74.4 to 87.0	NS
Testing of assumptions	13	139	9.4	5.5 to 15.4	34	155	21.9	16.1 to 29.1	NS	NS	110	6.4	3.1 to 12.8	9	133	6.8	3.6 to 12.5	NS
Reporting measures of variation	112	149	75.2	67.6 to 81.4	132	162	81.5	74.8 to 86.7	NS	89	125	71.2	62.7 to 78.4	103	147	70.1	62.1 to 76.9	NS
Variation less than twofold	9	40	18.4	9.8 to 31.7	15	55	27.3	17.2 to 40.4	NS	12	46	26.1	15.4 to 40.5	17	45	37.8	24.9 to 52.6	NS

n, number meeting the criteria; N, total number of studies where that criteria is relevant; %, per cent meeting the criteria; CI, 95% CI of that percentage; Adj p, adjusted significance level (two-sample proportion test (prop.test) followed by Holm-Bonferroni correction (p.adjust.methods)); NS, not significant at p<0.05.

NPG, Nature Publishing Group.

### Other checklist items

For reporting of details of animals used, reporting of animal species and strain was high even before the change in editorial policy. There was no significant change in reporting any of these items in NPG and non-NPG articles, or in the reporting of details of antibodies used. For in vitro research, there was an increase in the proportion of studies in NPG articles reporting recent mycoplasma testing of the cell lines used (from 1% to 26%, Χ²=26.60, df=1, adjusted p=4×10^−5^) but not for non-NPG articles (1% before, 1% after). For reporting and availability of accession data (eg, DNA or protein sequence deposition) and computer code, there were no significant changes for either NPG or non-NPG articles. Finally, there were no significant changes in the reporting of items relating to human subjects or the use of human materials, but for most items the number of articles for which these were relevant was very low indeed (table 7).

**Table 7:** Other secondary outcomes

Item	NPG before				NPG after				Adj p	Matched before				Matched after				Adj p
	n	N	%	CI	n	N	%	CI		n	N	%	CI	n	N	%	CI	
Animals																		
Was the species reported?	203	203	100.0	97.7 to 100	189	189	100.0	97.5 to 100	NS	163	164	99.4	95.8 to 99.9	188	189	99.5	96.3 to 99.9	NS
Was the strain reported?	187	203	92.1	87.5 to 95.1	181	189	95.8	91.8 to 97.9	NS	149	164	90.9	85.4 to 94.4	176	189	93.1	88.5 to 96.0	NS
Was the sex reported?	69	193	35.8	29.3 to 47.8	96	184	52.2	45.0 to 59.3	NS	59	161	36.6	29.6 to 44.4	67	183	36.3	30.0 to 43.8	NS
Was exact age or weight given?	31	203	15.3	11.0 to 20.9	41	188	21.8	16.5 to 28.3	NS	41	164	25.0	19.0 to 32.2	39	189	20.6	15.4 to 27.0	NS
Was ethical approval reported?	116	194	59.8	52.7 to 66.5	121	178	68.0	60.1 to 74.4	NS	91	151	60.3	52.3 to 67.8	123	180	68.3	61.2 to 74.7	NS
Ethical guidelines reported?	148	199	74.4	67.9 to 80.0	154	181	85.1	79.1 to 89.6	NS	111	151	73.5	65.9 to 79.9	137	181	75.7	68.9 to 81.4	NS
Reagents																		
In vivo antibodies	89	142	62.7	54.4 to 70.2	98	135	72.6	64.5 to 79.4	NS	38	115	33.0	25.1 to 42.1	58	127	45.7	37.2 to 54.4	NS
In vitro antibodies	75	125	60.0	51.2 to 68.2	107	142	75.4	67.6 to 81.7	NS	29	104	27.9	20.1 to 37.2	51	126	40.5	32.2 to 49.2	NS
Total antibodies	164	267	61.4	55.4 to 67.1	205	277	74.0	68.5 to 78.8	NS	67	219	30.6	24.8 to 37.0	109	253	43.1	37.1 to 49.3	NS
Cell lines																		
Cell line source	51	102	50.0	40.4 to 59.6	96	137	70.1	61.9 to 77.1	NS	53	84	63.1	52.3 to 72.7	62	111	55.9	46.5 to 64.8	NS
Recent authentication?	1	95	1.1	0.2 to 7.1	9	126	7.1	3.8 to 13.2	NS	4	76	5.3	2.0 to 13.2	1	97	1.0	0.2 to 7.0	NS
Recent mycoplasma testing?	1	97	1.0	0.2 to 7.0	33	127	26.0	19.1 to 34.3	4×10^−5^	1	77	1.3	0.2 to 8.6	1	97	1.0	0.2 to 7.0	NS
Data deposition																		
Accession: DNA/protein	30	61	49.2	36.9 to 61.5	32	64	50.0	38.0 to 62.0	NS	10	21	47.6	27.8 to 68.2	19	45	42.2	28.8 to 56.9	NS
Accession: macromolecular	0	4	0	0.0 to 60.4	4	7	57.1	20.2 to 88.2	NT	0	2	0	0.0 to 80.2	3	4	75.0	21.9 to 98.7	NT
Accession: crystallography	5	7	71.4	30.2 to 94.9	3	11	27.3	7.3 to 60.7	NT	1	2	50.0	9.4 to 90.5	0	1	0	0.0 to 94.5	NT
Accession: microarray	12	33	36.4	21.9 to 53.7	21	38	55.3	39.5 to 70.1	NS	7	15	46.7	24.1 to 70.7	12	18	66.7	42.9 to 84.2	NS
Accession: other	2	7	28.7	7.2 to 67.3	8	18	44.4	24.0 to 67.0	NT	1	5	20.0	10.5 to 70.1	4	6	66.7	24.1 to 94.0	NT
Code																		
Computer code with paper?	3	14	21.4	7.1 to 49.4	5	24	20.8	9.0 to 41.3	NS	0	5	0	0.0 to 53.7	2	14	14.3	3.6 to 42.7	NT
Code in public domain?	3	11	27.3	9.0 to 58.6	5	24	20.8	9.0 to 41.3	NS	0	5	0	0.0 to 53.7	2	13	15.4	3.9 to 45.1	NT
Was that code accessible?	2	3	66.7	12.5 to 98.2	5	7	71.4	30.2 to 94.9	NT	0	1	0	0.0 to 94.5	2	4	50.0	15.0 to 85.0	NT
Did the code function?	2	3	67.7	12.5 to 98.2	2	2	100.0	19.8 to 100	NT		0				0			NT
Say where you could get code?	0	9	0	0.0 to 37.1	1	16	6.3	0.3 to 32.3	NT	2	5	40.0	7.2 to 83.0	0	10	0	0.0 to 34.4	NT
Human materials																		
Reporting ethical approvals	35	43	81.4	67.0 to 90.4	46	56	82.1	69.9 to 90.1	NS	13	23	56.5	36.3 to 74.8	29	38	76.3	60.4 to 87.2	NS
Reporting consent	35	42	83.3	69.0 to 91.8	47	56	83.9	71.9 to 91.4	NS	15	24	62.5	42.2 to 79.2	24	38	63.2	47.0 to 76.8	NS
Consent to photos	2	3	66.7	12.5 to 98.2	1	1	100.0	5.4 to 100	NT		0			0	2	0	0.0 to 80.2	NT
Clinical trial number		0			2	3	66.7	12.5 to 98.2	NT	1	1	100.0	5.4 to 100		0			NT
CONSORT		0				0			NT	0	1	0	0.0 to 94.5		0			NT
REporting recommendations for tumour MARKer prognostic studies (REMARK)	0	1	0	0.0 to 94.5		0			NT	0	2	0	0.0 to 80.2	0	1	0	0.0 to 94.5	NT

n, number meeting the criteria; N, total number of studies where that criteria is relevant; %, per cent meeting the criteria; CI, 95% CI of that percentage; Adj p, adjusted significance level (two-sample proportion test (prop.test) followed by Holm-Bonferroni correction (p.adjust.methods)); NS, not significant at p<0.05; NT, not tested (n<10 for one of the comparisons).

CONSORT, Consolidated Standards of Reporting Trials; NPG, Nature Publishing Group.

In our protocol we defined the smallest effect size of editorial interest following the intervention at NPG as either achievement of compliance of 80% (transparency in figures and statistical description, data deposition, and for in vivo research description of animals used and aggregate and individual compliance Landis 4 items) or an absolute improvement of 15% in the reporting of a checklist item (all other items). Table 8 shows, for each item, the 95% CI of both the compliance achieved and the change in compliance before and after checklist implementation. Using this approach, we were able reliably to exclude an improvement of 15% or more in NPG manuscripts for full and individual item Landis compliance for in vitro research; reporting full statistics for both in vivo and in vitro research; correction for multiple testing, and reporting measures of variation, and whether the exact age or weight was given for in vivo research; and reporting whether the central estimate was mean or median, and whether cell lines used had recently been authenticated for in vitro research.

**Table 8:** 95% CIs for the observed compliance (columns 1–4) and change in compliance (columns 5 and 6)

Item	95% CI for compliance				95% CI for change in compliance	
	NPG before	NPG after	Non-NPG before	Non-NPG after	NPG	Non-NPG
In vivo: full Landis	–	11.7 to 22.2	0.0 to 4.2	0.0 to 3.7	11.1 to 21.6	−1.6 to 1.5
In vitro: full Landis	–	1.5 to 7.1	–	0 to 4.2	0.7 to 5.9	−0.6 to 1.8
In vivo: randomisation	6.3 to 15.5	60.9 to 74.8	3.0 to 11.5	7.2 to 17.7	49.1 to 65.7	−1.3 to 12.3
In vivo: blinding	3.1 to 9.8	55.3 to 69.2	0.6 to 5.6	3.3 to 10.4	48.7 to 64.0	−0.2 to 8.6
In vivo: exclusions reported	9.7 to 19.3	24.5 to 37.6	6.1 to 15.3	10.1 to 20.0	8.6 to 24.8	−2.3 to 11.3
In vivo: sample size calculation	1.7 to 7.3	57.1 to 70.9	0.0 to 4.4	1.8 to 7.7	52.7 to 67.6	−0.3 to 7.0
In vitro: randomisation	–	12.9 to 24.4	0.1 to 5.5	–	12.3 to 24.3	−4.4 to 1.8
In vitro: blinding	2.2 to 9.2	17.2 to 29.6	0.7 to 6.9	2.0 to 8.5	11.2 to 25.5	−2.8 to 6.4
In vitro: exclusions reported	4.8 to 13.6	11.3 to 22.0	2.5 to 10.6	4.6 to 12.9	0.1 to 14.6	−3.6 to 8.2
In vitro: sample size calculation	2.2 to 9.2	27.8 to 41.8	–	2.0 to 8.5	22.0 to 37.6	0.6 to 8.4
In vivo: exact n	39.8 to 52.7	62.4 to 75.5	38.8 to 54.0	40.1 to 54.2	13.8 to 32.6	−9.6 to 11.0
In vivo: technical or biological replicates	61.4 to 74.2	74.0 to 85.4	48.4 to 63.5	47.1 to 61.2	3.4 to 20.6	−12.1 to 8.5
In vivo: number of times replicated	17.3 to 28.9	30.5 to 44.4	15.7 to 28.3	14.1 to 25.4	5.4 to 23.5	−10.7 to 6.2
In vivo: test described if uncommon?	37.5 to 64.3	48.3 to 71.7	41.1 to 71.9	42.4 to 69.9	−8.4 to 26.4	−19.7 to 18.9
In vivo: t-test defined as one-sided or two-sided?	37.6 to 54.4	62.9 to 77.7	21.8 to 39.7	36.8 to 55.3	13.4 to 35.6	2.7 to 28.2
In vivo: correction for multiplicity	45.2 to 63.1	47.6 to 65.1	46.6 to 65.8	53.7 to 71.0	−10.2 to 14.6	−6.6 to 19.0
In vivo: reporting full statistics	17.6 to 30.4	15.6 to 27.8	10.3 to 22.5	8.4 to 19.0	−6.6 to 10.6	−10.9 to 5.4
In vivo: reporting of average	67.7 to 80.4	79.1 to 90.4	67.2 to 81.2	64.8 to 78.3	3.0 to 19.5	−12.4 to 7.0
In vivo: definition of error bars	82.2 to 91.9	87.1 to 95.4	74.8 to 87.4	71.2 to 83.8	−0.12 to 10.8	−12.5 to 5.3
In vivo: testing of assumptions	5.4 to 14.0	20.5 to 33.9	5.2 to 15.1	7.4 to 17.6	9.8 to 25.9	−4.7 to 9.6
In vivo: reporting measures of variation	71.6 to 83.5	74.2 to 86.0	73.4 to 86.9	66.6 to 80.3	−5.8 to 11.0	−16.1 to 2.4
In vivo: variation less than twofold	14.8 to 37.8	25.1 to 48.8	14.5 to 38.4	22.3 to 48.0	−5.4 to 27.3	−8.3 to 26.5
In vitro: exact n	25.4 to 40.0	44.1 to 58.6	29.1 to 45.4	19.9 to 33.3	8.6 to 29.0	−21.2 to −0.2
In vitro: technical or biological replicates	48.8 to 64.1	68.5 to 81.0	31.2 to 47.6	35.1 to 50.1	8.6 to 28.3	−7.9 to 14.3
In vitro: number of times replicated	31.9 to 47.1	39.8 to 54.2	28.6 to 44.9	28.4 to 43.0	−2.8 to 18.0	−11.9 to 9.8
In vitro: test described if uncommon?	39.7 to 69.0	49.0 to 73.6	29.5 to 58.0	22.6 to 51.9	−11.7 to 26.0	−26.9 to 13.4
In vitro: t-test defined as one-sided or two-sided?	37.6 to 56.2	64.0 to 79.3	23.6 to 43.8	24.7 to 44.1	13.1 to 37.0	−13.2 to 14.6
In vitro: correction for multiplicity	37.6 to 56.7	42.6 to 60.8	36.7 to 57.6	44.2 to 63.5	−8.5 to 17.7	−7.4 to 20.9
In vitro: reporting full statistics	12.4 to 25.1	11.8 to 23.5	8.9 to 22.0	9.2 to 21.0	−9.7 to 7.6	−9.3 to 8.5
In vitro: reporting of average	74.5 to 87.3	81.4 to 91.8	62.9 to 78.6	67.4 to 81.3	−2.4 to 13.8	−6.8 to 14.1
In vitro: definition of error bars	85.6 to 94.9	89.8 to 97.1	72.3 to 86.4	74.4 to 87.0	−2.6 to 9.4	−8.2 to 10.8
In vitro: testing of assumptions	5.5 to 15.4	16.1 to 29.1	3.1 to 12.8	3.6 to 12.5	4.2 to 20.7	−6.6 to 6.9
In vitro: reporting measures of variation	67.6 to 81.4	74.8 to 86.7	62.7 to 78.4	62.1 to 76.9	−2.8 to 15.5	−11.8 to 9.7
In vitro: variation less than twofold	9.8 to 31.7	17.2 to 40.4	15.4 to 40.5	24.9 to 52.6	−7.5 to 24.3	−7.3 to 30.0
Animals: was the species reported?	97.7 to 100	97.5 to 100	95.8 to 99.9	96.3 to 99.9	−2.0 to 1.9	−2.4 to 2.9
Animals: was the strain reported?	87.5 to 95.1	91.8 to 97.9	85.4 to 94.4	88.5 to 96.0	−1.2 to 8.6	−3.5 to 8.4
Animals: was the sex reported?	29.3 to 47.8	45.0 to 59.3	29.6 to 44.4	30.0 to 43.8	6.4 to 26.0	−10.2 to 10.0
Animals: was exact age or weight given?	11.0 to 20.9	16.5 to 28.3	19.0 to 32.2	15.4 to 27.0	−1.2 to 14.3	−13.2 to 4.4
Animals: was ethical approval reported?	52.7 to 66.5	60.1 to 74.4	52.3 to 67.8	61.2 to 74.7	−1.6 to 17.7	−2.2 to 18.3
Animals: ethical guidelines reported?	67.9 to 80.0	79.1 to 89.6	65.9 to 79.9	68.9 to 81.4	2.6 to 18.6	−7.1 to 11.6
Reagents: in vivo antibodies	54.4 to 70.2	64.5 to 79.4	25.1 to 42.1	37.2 to 54.4	−1.1 to 20.6	0.2 to 24.4
Reagents: in vitro antibodies	51.2 to 68.2	67.6 to 81.7	20.1 to 37.2	32.2 to 49.2	4.1 to 26.2	0.2 to 24.2
Reagents: total antibodies	55.4 to 67.1	68.5 to 78.8	24.8 to 37.0	37.1 to 49.3	4.7 to 20.2	3.8 to 20.9
Cell line: source	40.4 to 59.6	61.9 to 77.1	52.3 to 72.7	46.5 to 64.8	7.6 to 31.9	−20.5 to 6.7
Cell line: recent authentication?	0.2 to 7.1	3.8 to 13.2	2.0 to 13.2	0.2 to 7.0	0.3 to 12.0	−11.8 to 1.4
Cell line: recent mycoplasma testing?	0.2 to 7.0	19.1 to 34.3	0.2 to 8.6	0.2 to 7.0	16.7 to 33.2	−6.0 to 4.4
Accession: DNA/protein	36.9 to 61.5	38.0 to 62.0	27.8 to 68.2	28.8 to 56.9	−16.2 to 17.8	−29.4 to 18.7
Accession: macromolecular	0.0 to 60.4	20.2 to 88.2	0.0 to 80.2	21.9 to 98.7	−1.4 to 84.1	−4.6 to 95.4
Accession: crystallography	30.2 to 94.9	7.3 to 60.7	9.4 to 90.5	0.0 to 94.5	−71.0 to 1.9	−39.1 to 90.6
Accession: microarray	21.9 to 53.7	39.5 to 70.1	24.1 to 70.7	42.9 to 84.2	−4.2 to 39.2	−12.6 to 47.7
Accession: other	7.2 to 67.3	24.0 to 67.0	10.5 to 70.1	24.1 to 94.0	−24.8 to 45.7	−9.4 to 75.4
Computer code: with paper?	7.1 to 49.4	9.0 to 41.3	0.0 to 53.7	3.6 to 42.7	−29.2 to 23.4	−30.4 to 39.9
Computer code: in public domain?	9.0 to 58.6	9.0 to 41.3	0.0 to 53.7	3.9 to 45.1	−37.9 to 19.9	−29.4 to 42.2
Computer code: was that code accessible?	12.5 to 98.2	30.2 to 94.9	0.0 to 94.5	15.0 to 85.0	−40.0 to 55.0	−36.7 to 85.0
Computer code: did the code function ?	12.5 to 98.2	19.8 to 100	–	–	−37.8 to 79.2	–
Computer code: say where you could get code?	0.0 to 37.1	0.3 to 32.3	7.2 to 83.0	0.0 to 34.4	−24.1 to 28.3	−76.9 to −0.4
Human materials reporting ethical approvals	67.0 to 90.4	69.9 to 90.1	36.3 to 74.8	60.4 to 87.2	−14.2 to 16.8	−3.9 to 42.2
Human materials reporting consent	69.0 to 91.8	71.9 to 91.4	42.2 to 79.2	47.0 to 76.8	−13.8 to 16.4	−22.1 to 24.6
Human materials consent to photos	12.5 to 98.2	5.4 to 100		0.0 to 80.2	−50.5 to 79.2	–
Human materials clinical trial number		12.5 to 98.2	5.4 to 100		–	–
Human materials CONSORT			0.0 to 94.5		–	–
Human materials REMARK	0.0 to 94.5		0.0 to 80.2	0.0 to 94.5	–	−65.8 to 79.4

Colours represent CIs falling below (red), encompassing (yellow) and exceeding (green) 80% compliance (1–4) or a 15% improvement in compliance. Deeper colours relate to the editorially important change criteria identified in the study protocol. Grey boxes: not applicable.

CONSORT, Consolidated Standards of Reporting Trials; NPG, Nature Publishing Group.

**Figure 3:**
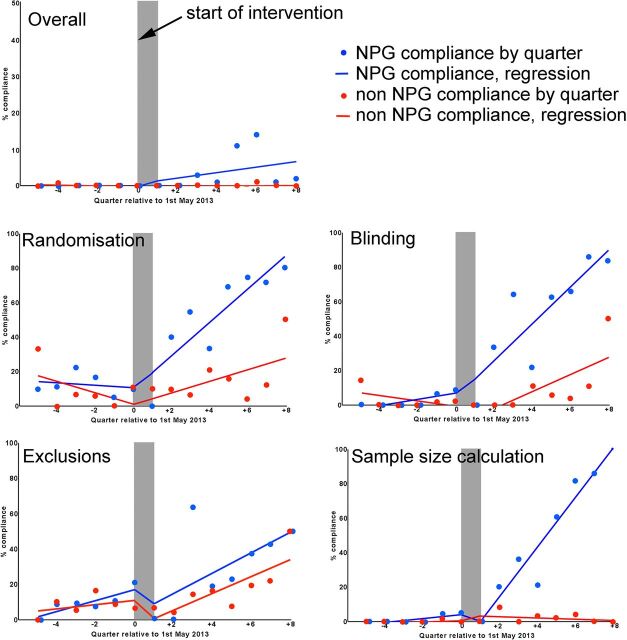
Interrupted time series analysis for overall Landis compliance and compliance with Landis components in in vivo experiments reported in NPG and non-NPG articles overall, and individually for randomisation, blinding, reporting of animals excluded from analysis and sample size calculations. NPG, Nature Publishing Group.

**Figure 4:**
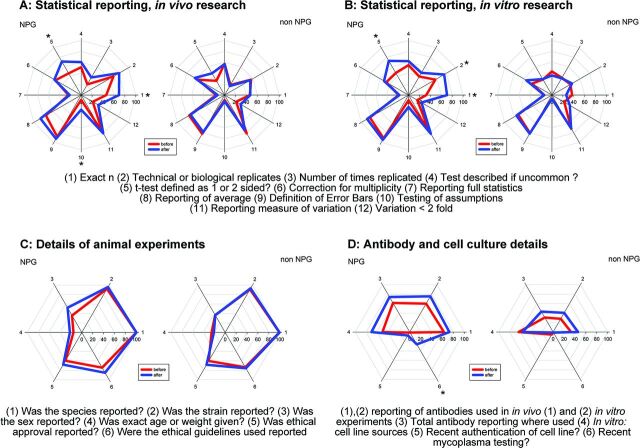
Radar plots for compliance with individual components of the NPG checklist before (red) and after (blue) 1 May 2013 for (A) statistical reporting, in vivo research; (B) statistical reporting, in vitro research; (C) reporting of details of animals used; and (D) reporting of reagents used. *Adjusted p<0.05 for change between ‘before’ and ‘after’. NPG, Nature Publishing Group.

**Figure 5:**
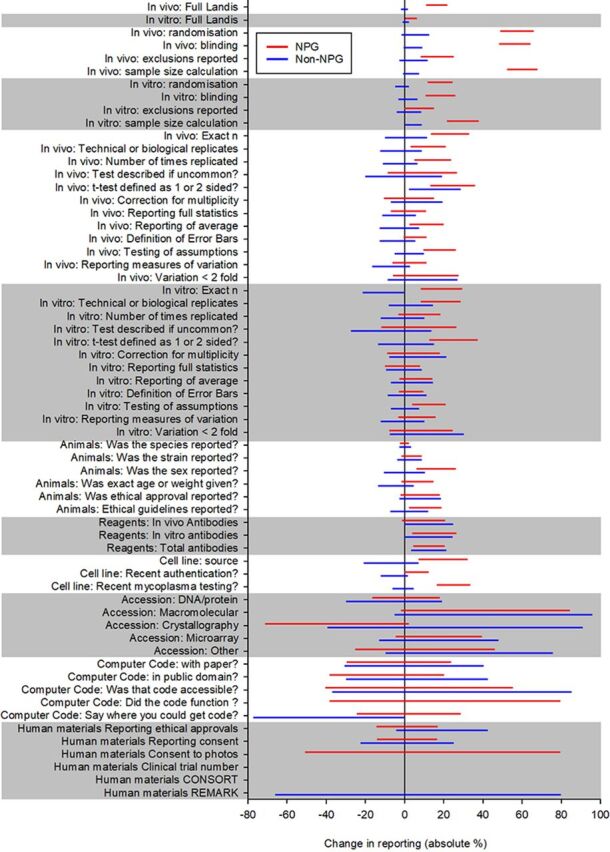
95% CIs, for each item, for the change in performance in NPG (red) and non-NPG (blue) cohorts. CONSORT, Consolidated Standards of Reporting Trials; NPG, Nature Publishing Group.

We were also interested in whether changes in reporting had occurred as a step change at the time of the change in editorial policy; whether there was an initial improvement with then a return to previous performance; or if there was an ongoing improvement in reporting. To address this question we conducted an interrupted time series analysis to estimate the rate of change before the intervention, any step change at the time of the intervention and the rate of change after the intervention. We grouped articles in 3-month periods starting November 2011, and for each quarter calculated the proportional compliance with the criteria in question. Because articles were not evenly distributed across time, the analysis is of substantially reduced power, but the fitted lines for overall compliance and for each component of the Landis checklist for in vivo research are shown in figure 3. It appears that with the exception of sample size calculation, there is a continuing improvement over time in both NPG and non-NPG articles; for sample size calculations, the improvement is only seen in NPG articles. Figure 4 shows radar charts of compliance for each checklist item in NPG and non-NPG articles before and after May 2013. Figure 5 shows the 95% CIs for the change in performance for each checklist item.

## Discussion

The change in editorial policy at NPG was associated with improvements in reporting of randomisation, blinding, exclusions from analysis and sample size calculations. For the highly challenging primary outcome measure, full compliance increased from 0% to 16%. This falls short of the target compliance of 80%, but should be seen in the context, first, that only 1 of 1073 articles from 2009 to 10 from leading UK institutions achieved this standard,^1^ and second that overall compliance of 80% would require compliance with individual items of around 95%. Since these results were first communicated at the Peer Review Congress, in bioRxiv and in the peer review process, it has been suggested that the observed change is small. It certainly falls well short of the target of 80% compliance, but this needs to be seen in the context, first, of the generally poor reporting of biomedical research (as evidenced in the non-NPG cohort) and in the challenge involved with achieving compliance for a composite outcome. If the probabilities of compliance with each of the Landis 4 items were independent of each other, then achieving 80% compliance overall would require compliance with individual items of 94%.

Prior to the study we identified achievement of 80% compliance, or an absolute improvement of 15% in the reporting of an item, as being the minimal change which would represent an important effect of an editorial intervention. In the NPG cohort, for 62 items the 95% CIs of the observed change fell below 15% for 11 items, included 15% for 40 items and were above 15% for 5 items. For three items there were insufficient data to calculate 95% CIs, and for three items baseline performance already exceeded 85%.

Power calculations in primary research are often considered unfeasible, on the basis that prior to doing the study the effect size is not known. Our approach here—of identifying a smallest effect size of interest—is increasingly widely used, and has allowed us to demonstrate if any change observed might be as large as the smallest effect size of interest, is definitely that large or is definitely not that large. We hope that those using our findings to guide their own improvements will find this helpful and recommend the approach for use in future studies.

It is notable that even with considerable investment in designing and implementing a checklist, and working with authors to encourage its completion, compliance remains so low. This stands rather in contrast to the belief that ‘all’ that is required to ensure transparency in reporting is that journals ‘insist’ that authors do the right thing. Securing transparency in research reports is a complex challenge, and experience in other fields (MM is also clinical lead for a clinical neurology service) suggests such challenges require a range of complementary approaches with commitment from all stakeholders, might best be achieved through formal improvement activity, and often take multiple attempts to achieve and sustain change.

The checklist relates to transparency in reporting, and articles were judged to be compliant if they either reported measures to address that risk of bias or reported that such measures were not taken. For each of the Landis 4 criteria, compliance was most often achieved by the authors reporting that they had not taken measures to reduce the risk of bias. While this is not ideal, we believe this represents an improvement, in terms of the usefulness of the research to those who wish to use it, from a situation where these issues are not reported at all.

For reports of in vivo research, compliance for randomisation, blinding, reporting of exclusions and sample size calculations in NPG articles reached 68%, 63%, 31% and 64%, respectively. For non-NPG articles the performance was 12%, 5%, 12% and 3%. The figures for NPG articles are similar to those recently reported for in vivo research published in the journal *‘Stroke’*,^13 14^ which began requiring reporting of such details following the publication of good practice guidelines in 2009,^15^ and where performance was found to be substantially higher than for in vivo research published in other American Heart Association journals.^14^

While we saw improvements in the transparency of reporting, the observed improvements in experimental design were much more modest. However, peer review may not ensure the quality of published work,^16^ as evidenced for in vivo research by poor reporting of measures to reduce risks of bias.^1^ We believe that the ultimate responsibility for assessing research quality (and therefore the validity of the findings presented) rests with the reader, and transparency in reporting is fundamental to this assessment.

For reports of in vitro research, compliance was substantially lower. There have been few systematic attempts to measure the quality of reporting of measures to reduce the risks of bias in vitro research, and our findings suggest that, both in NPG and non-NPG journals, this remains low. There were improvements in reporting randomisation, blinding and sample size calculations in NPG descriptions of in vitro research, but only to 18%, 23% and 34%, respectively. For non-NPG the equivalent figures were 3%, 1% and 1%. There were no significant changes in the reporting of exclusion of in vitro data, with postintervention compliance of 16% in NPG articles and 6% in non-NPG articles.

For other checklist items, changes in performance were less dramatic, but there appeared to be incremental improvements across most of the items measured, although few of these breached our rather parsimonious adjustment for multiple testing. In spite of substantial attention given to the importance of reporting the sex of experimental animals, this was only done in 52% of postintervention NPG studies and in 36% of non-NPG studies.

Our assessment of compliance with the checklist was based on the resulting manuscript, and not on the completed checklists submitted by the study authors, which were not available to us for analysis. Therefore, we do not know whether these submitted checklists were incomplete but the requirement for compliance was not completely enforced, or if the authors and editors considered that manuscripts were compliant but our outcome assessors disagreed with those judgements. Knowing the relative contribution of these two explanations would inform refinements to checklist-based strategies to improve reporting. Of note, for the Consolidated Standards of Reporting Trials checklist, Blanco and colleagues recently showed that the checklist as submitted was concordant with the manuscript as published for only one of six studies.^1^

Ours is an observational study, and it is possible that other (related or unrelated) changes were responsible for much if not all the differences seen. These changes were not observed in other journals (at least not when taken in aggregate), and so it is likely that alternative causal factors would relate to NPG editorial policy and practice. While we are not aware of any other relevant changes in editorial policy occurring at a relevant time, it is likely that this change in editorial policy was accompanied by increased attention given to the importance of the quality of reporting by both inhouse editorial staff and external peer reviewers. It is not possible to determine whether these might have caused the changes seen. However, a randomised controlled study of the effect of the Animal Research: Reporting of In Vivo Experiments (ARRIVE) checklist completion on the quality of reporting of in vivo research at *PLoS One* will report shortly.

While our primary outcome measure was unchanged, when writing our data analysis plan (and prior to any data inspection or analysis), we did change our criterion for measuring success, from ‘whether compliance (with the Landis 4 criteria, for in vivo research) in the postintervention group of articles reached 80%’ to ‘the change in proportion of articles describing in vivo research meeting the 4 Landis criteria’. This was because our primary intention had been to observe any effect of a change in publication policy, and with the benefit of hindsight this was not captured in our original primary outcome, but we recognise this as a limitation in our findings. We note, however, that the primary outcome used reflects better the title of the study protocol than does the primary outcome measure proposed in that protocol.

For our comparator group we chose similar articles with a similar date of publication identified using the PubMed ‘related citations’ tool. The journals in which these works were published will vary in the attention which they have given to transparency in reporting, and it may be that for some journals there have been changes similar to those observed in the NPG articles. While we might have restricted our comparator group to journals more similar to NPG articles (for instance by impact factor or extent of editorial intervention), this would have meant lower fidelity of matching by subject area or date of publication or both, and we considered these factors to be more important. For this reason, our findings for NPG articles cannot be interpreted as showing improved reporting compared with similar articles in similar journals. The representation of such ‘similar’ journals in the comparator group is too small to allow meaningful conclusions to be drawn.

During the study we encountered some difficulties that we had not expected. We had thought that it would be straightforward to distinguish between an in vivo experiment and an in vitro experiment, but we had to develop an operational approach which defined that experiment on the basis of the subject at the time that the experimental intervention occurred; so a tissue slice experiment involving tissues from animals exposed to treatment or control we considered in vivo, while a similar experiment applying drugs directly to the slice we considered to be an in vitro experiment.

Our matching on whether studies reported in vitro or in vivo research or both was also reasonable in most cases. Differences will have emerged where, as described above, articles were initially categorised with one set of characteristics (in vitro, in vivo or both) and matched accordingly, but later judged to have different characteristics. Our matching for date of publication worked reasonably well, apart from the inclusion of one comparator article published in 1995, 215 months before its ‘matching’ NPG article. We had not anticipated that matching articles would be so difficult to identify, so our matching rules did not have an upper limit of difference in the date of publication. An alternative approach would have been to prioritise matching on data of publication rather than manuscript content, but each approach has its weaknesses. Because one group (non-NPG studies published before May 2013) is substantially smaller, this will have limited, to an extent, the statistical power of these contrasts; however, since power changes with the square root of the number of studies, we estimate this loss of power only to be around 10%. Since the comparator (non-NPG) group does not contribute to our primary outcome, and the matching is generally good, we do not think that these mismatches devalue our findings to any appreciable extent.

Our matching by country of origin in the NPG cohort of publications may have introduced a bias in that manuscripts from countries with fewer publications may have been excluded because of the lack of an appropriate match. However, the included manuscripts had a country of origin matching more than 85% of Nature papers published between 2010 and 2016, and so it is unlikely that this has introduced major bias.

Further, there were some checklist items where agreement between outcome assessors was very low—for instance, for the question of whether for in vivo research the difference in variance between groups being compared was less than twofold, the agreement was no better than would be expected by chance alone. We recommend that the future development of publication checklists should include an assessment of interobserver variation by potential users of the checklist for each checklist item; low agreement might indicate that the item should be rephrased or reframed, or that more explanatory text is required.

We encountered a further unexpected problem when assessing compliance with reporting of blinding, randomisation and with sample size calculations. These were assessed with pairs of questions: first did the study report doing it (yes/no/not relevant); and second did they at least mention it (yes/no/not relevant). If a study was ‘yes’ for the first question, assessors were instructed to score the second as not relevant. Therefore, the number scored as ‘not relevant’ for the second question should represent the sum of those scored as ‘yes’ and as ‘not relevant’ for the first. This was not always the case (for in vivo research occurring in 0.1%, 0.8% and 6% of assessments for sample size calculation, randomisation and blinding, respectively), but we did not become aware of this problem until after database lock. Any impact of this shortcoming is likely to be small.

Finally, our work shows the challenge of assessing even a relatively limited number of articles against a relatively straightforward checklist. We are delighted that so many collaborators (from six continents) agreed to participate and are very grateful to them. However, even with their help the outcome assessment and reconciliation took 17 months. This is too slow to be useful, for instance, for quality improvement activity, where more rapid feedback would allow more rapid adjustments in response to performance. We have tested the use of text analytics using regular expressions to automatically ascertain reporting of measures to reduce the risk of bias, and for some such risks of bias the approach achieves sensitivities and specificities above 80%.^17^ For more complex items it is likely that machine learning approaches using, for instance, convoluted neural networks may be more successful, and this is a current focus of our research. We hope that, by making the data set for this study available, this might be used, for instance, for distant supervised learning in such systems. However, the extent of disagreement between our trained assessors suggests that the language used to describe experiments in biomedicine is not altogether clear, and both machines and human may require greater clarity in reporting to fully understand published research.

## Conclusions

Introduction of a checklist leads to substantial improvements in the quality of reporting in NPG articles that were not seen in matched articles from other publishers, and these improvements appear to be ongoing. However, there is still substantial room for improvement, which suggests that measures such as mandatory author checklists need to be supplemented by other approaches.
